# A Treatise on Materia Medica, Pharmacology, and Therapeutics

**Published:** 1891-03

**Authors:** 


*A Treatise on Materia Medica, Pharmacology, and Therapeutics.* By J. V. SHOEMAKER, A.M., M.D., and JOHN AULDE, M.D. Vol. I. Philadelphia: F. A. DAVIS. 1889.

The names of Drs. Shoemaker and Aulde are a guarantee that their joint product will be worth possession, and still more worth consulting as a guide. This first volume does not deal with Materia Medica in the ordinary sense of the term, but with remedial agents not properly classed as drugs, and thereby fills a large lacuna in subjects treated of in most works upon Materia Medica published in this country. The list of remedial agents described and discussed is one of unusual completeness and extent, including such topics as earth-dressing, baunscheidtismus, and music. Electro-therapeutics are discussed in much detail, as is customary in American works on therapeutics or medicine; and we get a little fine writing on this favourite topic. For example, "What a powerful agent is this, capable of dynamic and chemical effects, with which we can touch as with the thrill from the tip of an angel's wing or blast with the fire of the lowest abyss!" &c., &c. "This is lofty," as Bottom says, but still the book is solidly useful.

